# Distributed Piezoelectric Sensor System for Damage Identification in Structures Subjected to Temperature Changes

**DOI:** 10.3390/s17061252

**Published:** 2017-05-31

**Authors:** Jaime Vitola, Francesc Pozo, Diego A. Tibaduiza, Maribel Anaya

**Affiliations:** 1 Control, Dynamics and Applications (CoDAlab), Departament de Matemàtiques, Escola d’Enginyeria de Barcelona Est (EEBE), Universitat Politècnica de Catalunya (UPC), Campus Diagonal-Besòs (CDB), Eduard Maristany, 6–12, Sant Adrià de Besòs (Barcelona) 08930, Spain; jaimevitola@usantotomas.edu.co; 2 MEM (Modelling-Electronics and Monitoring Research Group), Faculty of Electronics Engineering, Universidad Santo Tomás, Bogotá 110231, Colombia; 3 Departamento de Ingeniería Eléctrica y Electrónica, Universidad Nacional de Colombia, Cra 45 No. 26-85, Bogotá 111321, Colombia; dtibaduizab@unal.edu.co; 4 Facultad de Ingeniería, Fundación Universitaria Los Libertadores, Carrera 16 No. 63A-68, Bogotá 111221, Colombia; maribel.anaya@libertadores.edu.co

**Keywords:** machine learning, principal component analysis, piezoelectric sensors, temperature variations, damage classification

## Abstract

Structural health monitoring (SHM) is a very important area in a wide spectrum of fields and engineering applications. With an SHM system, it is possible to reduce the number of non-necessary inspection tasks, the associated risk and the maintenance cost in a wide range of structures during their lifetime. One of the problems in the detection and classification of damage are the constant changes in the operational and environmental conditions. Small changes of these conditions can be considered by the SHM system as damage even though the structure is healthy. Several applications for monitoring of structures have been developed and reported in the literature, and some of them include temperature compensation techniques. In real applications, however, digital processing technologies have proven their value by: (i) offering a very interesting way to acquire information from the structures under test; (ii) applying methodologies to provide a robust analysis; and (iii) performing a damage identification with a practical useful accuracy. This work shows the implementation of an SHM system based on the use of piezoelectric (PZT) sensors for inspecting a structure subjected to temperature changes. The methodology includes the use of multivariate analysis, sensor data fusion and machine learning approaches. The methodology is tested and evaluated with aluminum and composite structures that are subjected to temperature variations. Results show that damage can be detected and classified in all of the cases in spite of the temperature changes.

## 1. Introduction

The variability in the dynamic properties of a structure in service can be the result of time-varying environmental and operational conditions [1]. This variability is mainly one of the causes of an inaccurate damage identification process when the analysis of a structure is performed based on data-driven algorithms [2]. From this point of view, it is possible to affirm that the variability of environmental and operational conditions is one of the intrinsic features of the design of a structural health monitoring system [3].

There are many magnitudes to consider in the design of a structural health monitoring system; for instance, temperature, temperature gradients, humidity and wind or traffic [4]. When these factors are not considered, the results may mask or conceal the changes of the structure. Therefore, the diagnosis provided by the structural health monitoring system will not be accurate. For this reason, when designing a structural health monitoring system, it is very important to propose algorithms or methodologies that can cope with these environmental and operational conditions. The final goal is to offer an accurate damage identification process even improving the security of the structure and reducing the time and cost of its associated maintenance [5].

At present, it is possible to find some works in the literature that consider the effect of the environmental and operational variations. One of the most applied strategies to deal with these kinds of variations is principal component analysis (PCA). One of the main advantages of PCA is its ability to reduce the dimensionality of data, which is particularly useful when these data are collected from multiple sensors. In this sense, multivariate analysis has been proven to be effective for damage detection and classification [6,7]. In the same way, PCA is useful to perform linear analysis when it is assumed that the effect on the vibration features of the structure of the environmental conditions is linear or weakly non-linear [4]. PCA has also been used in combination with some other techniques or strategies. For instance, Torres–Arrendondo et al. [8] considers jointly discrete wavelet transform for feature extraction and selection, linear principal component analysis, for data-driven modeling, and self-organizing maps, for a two-level clustering under the principle of local density, for temperature compensation in acousto-ultrasonics. Leichtle et al. [9] apply principal component analysis jointly with *k*-means clustering for discrimination of changed and unchanged buildings as a method for unsupervised change detection in a dynamic urban environment. PCA has also been applied as a way to characterize the feature vector that defines the antigens and the antibodies in an artificial immune system conceived of to detect damage in structures under temperature variations [10]. The robust version of singular value decomposition (SVD), which is closely related to principal component analysis, has been used in [11] to compute the distance of an observation to the subspace spanned by the intact measurements. The distance to the subspace is therefore used to determine the presence of damage.

Structural health monitoring strategies that do not consider principal component analysis include, for instance, the work by Deraemaeker et al. [12], where the damage detection strategy is uniquely based on vibration measurements under changing environmental conditions. More precisely, two features are considered based on the measurements: the eigen-properties of the structure and peak indicators that are computed on the Fourier transform (FT) of modal filters. The effects of the changing environment are handled using factor analysis, and damage is detected by means of Shewhart-*T* control charts. Similarly, Balmès et al. [13] propose a nonparametric damage detection where it is assumed that several datasets are recorded on the safe structure at different and unknown temperatures. Finally, the approach smooths out the temperature effect using an averaging operation.

Buren et al. [14] address the damage detection problem combining three technologies to guarantee the robustness of a structural condition monitoring system subjected to environmental variability. One of these technologies is a time series algorithm that is trained with baseline data with three objectives: (a) to predict the vibration response; (b) to compare predictions to actual measurements collected on a damaged structure; and (c) to calculate a damage indicator. In this work [14], the robustness analysis is performed propagating the uncertainty through the time series algorithm and computing the equivalent deviation of the damage indicator.

Similar to PCA, time series analysis can also be combined with some other strategies, such as neural networks and statistical inference to develop damage classification strategies, including ambient variations of the system. For instance, Sohn et al. [15] developed an autoregressive and autoregressive with exogenous inputs (AR-ARX) model of the structure to extract damage-sensitive features, then used a neural network for data normalization and finally applied hypothesis testing to automatically infer the damage state of the system.

Several machine learning approaches have already been reported in the literature. For instance, it is possible to find the use of an auto-associative neural network (AANN), factor analysis, Mahalanobis distance and singular value decomposition [16] tested in a three-story frame structure where data are collected with accelerometers. Support vector machines (SVM) have also been applied for damage detection, localization and damage assessment in a Gnat trainer aircraft [17] showing the advantages in the use of machine learning approaches for damage identification. Some of these works use novelty detectors based on outlier analysis, density estimation and an auto-associative neural network [18,19] for these applications. Unsupervised machine learning algorithms and physics-based temperature compensation were also explored by Roy et al. [20]. More precisely, Roy et al. [20] use a neural network-based sparse autoencoder algorithm to learn the compressed representation of the data from sensors in order to localize damages in a structure with the Mahalanobis squared distance.

Previous works by the authors in this field include the use and development of multivariate analysis techniques, such as linear principal component analysis (PCA), non-linear PCA [7] and independent component analysis (ICA) to detect [21], classify and localize damage in structures [22]. In this paper, we present a structural health monitoring system based on [23] that is oriented to detect and classify the damage of a structure subjected to temperature variations. The system works with data collected from a piezoelectric sensor network attached permanently to the structure, and it introduces the use of a new way to organize the data, multivariate data analysis techniques and machine learning analysis. Some contributions of this system are the use of sensor data fusion, which introduces a different organization of the data, and the feature extraction vector for including temperature during the training process. This is a multivariate approach. This means that in the analysis, there are measurements from all of the sensors distributed all along the structure, which offers a generalized analysis from different points of view by fusing data in the only result. This procedure allows reducing the effect of the temperature in the damage detection and classification process when machine learning approaches are applied.

The structure of the paper is as follows: in Section 2, a brief description of the theoretical background required to construct the SHM system is presented. This background includes principal component analysis and machine learning approaches with special focus on how the three-way matrix with the collected data is unfolded to a two-way array. Section 3 describes the SHM system that is used to inspect the structures and the strategies that are applied to classify the damage in structures subjected to temperatures changes. In Section 4, the experimental setup is introduced together with exhaustive results. Finally, in Section 5, some concluding remarks are discussed.

## 2. Theoretical Background

### 2.1. Principal Component Analysis

One of the greatest difficulties in data analysis arises when the amount of data is very large and there is no apparent relationship between all of the information or when this relationship is very difficult to find. In this sense, principal component analysis was born as a very useful tool to reduce and analyze a big quantity of information. Principal component analysis was described for the first time by Pearson in 1901, as a tool of multivariate analysis and was also used by Hotelling in 1933 [24]. This method allows finding the principal components, which are a reduced version of the original dataset and include relevant information that identifies the reason for the variation between the measured variables. To find these variables, the analysis includes the transformation of the data with respect to a current coordinate space to a new space in order to re-express the original data trying to reduce, filter or eliminate the noise and possible redundancies. These redundancies are measured by means of the correlation between the variables [25].

There are two mechanisms to implement the analysis of principal components: (i) the first method is based on correlations; and (ii) a second strategy is based on the covariance. It is necessary to highlight that PCA is not invariant to scale, so the data under study must be normalized. Many methods can be used to perform this normalization, as is shown in [25,26]. In many applications, PCA is also used as a tool to reduce the dimensionality of the data. Currently, there are several useful toolboxes that implement PCA and analyze the reduced data provided by this strategy [27]. For the sake of completeness, the following sections present a succinct description of the PCA modeling that includes how the measured data are arranged in matrix form. We also present the normalization procedure (group scaling) and how the new data to inspect are projected onto the PCA model.

#### 2.1.1. PCA Modeling

**Figure 1:**
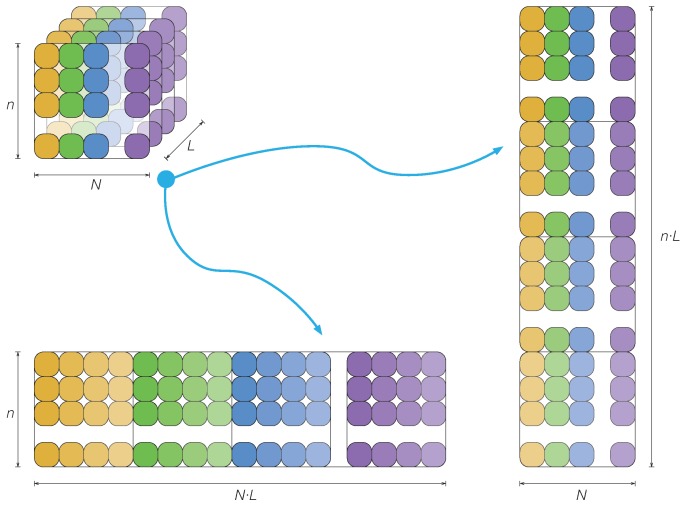
The three-way matrix Z can be unfolded to a two-way array in several ways.

As stated in Section 2.1, one of the considerable difficulties in data analysis emerges when the quantity of data is very large. In a general case, typical data from a batch process may consist of *N* variables measured at *L* time instants for *n* batches or experimental trials. These data can be easily arranged in a three-way matrix $Z\in M_{n\times N\times L}\left(R\right)$ as represented in Figure 1 (top, left). However, to apply multivariate statistical techniques, such as principal component analysis (PCA), this three-way matrix Z must be unfolded to a two-way array. Westerhuis et al. [28] discussed profoundly how to unfold this three-way matrix and what were the effects of data normalization on the multivariate statistical techniques. One of the possibilities that is presented in [28] is depicted in Figure 1 (right), where the three-way matrix $Z\in M_{n\times N\times L}\left(R\right)$ is unfolded to a two-way matrix with n·L rows and *N* columns. This way, each of the *N* columns in the unfolded matrix still represents the *N* variables that are measured in the process.

However, in our application, we propose a quite different approach to unfold the original three-way matrix Z. As can be observed in Figure 1 (bottom, left), the three-way matrix $Z\in M_{n\times N\times L}\left(R\right)$ is unfolded to a two-way matrix with *n* rows and N·L columns. This way, the columns of the unfolded matrix no longer represent the variables, but the measures of the variables at the different time instants. More precisely, the submatrix defined by taking the *n* rows and the first *L* columns represent the discretized measures of the first variable for the *n* batches or experimental trials; similarly, the submatrix defined by taking the *n* rows and columns L+1 to 2L represent the discretized measures of the second variable for the *n* batches or experimental trials. In general, then, the submatrix defined by taking the *n* rows and columns (l−1)·L+1 to l·L represents the discretized measures of the *l*-th variable for the *n* batches or experimental trials.

The first step to build a PCA model is to measure, from a healthy structure, different sensors or variables during (L−1)Δ seconds, where Δ is the sampling time, and n∈N experimental trials. The discretized measures of the sensors can be unfolded and arranged in matrix form as follows:

(1) $$\begin{array}{cc} X & =\left(\begin{array}{ccccccc} \begin{array}{cccc} x_{11}^{1} & x_{12}^{1} & \cdots & x_{1L}^{1}\\ \vdots & \vdots & \ddots & \vdots \\ x_{i1}^{1} & x_{i2}^{1} & \cdots & x_{iL}^{1}\\ \vdots & \vdots & \ddots & \vdots \\ x_{n1}^{1} & x_{n2}^{1} & \cdots & x_{nL}^{1} \end{array} & | & \begin{array}{ccc} x_{11}^{2} & \cdots & x_{1L}^{2}\\ \vdots & \ddots & \vdots \\ x_{i1}^{2} & \cdots & x_{iL}^{2}\\ \vdots & \ddots & \vdots \\ x_{n1}^{2} & \cdots & x_{nL}^{2} \end{array} & | & \begin{array}{c} \cdots \\ \ddots \\ \cdots \\ \ddots \\ \cdots \end{array} & | & \begin{array}{ccc} x_{11}^{N} & \cdots & x_{1L}^{N}\\ \vdots & \ddots & \vdots \\ x_{i1}^{N} & \cdots & x_{iL}^{N}\\ \vdots & \ddots & \vdots \\ x_{n1}^{N} & \cdots & x_{nL}^{N} \end{array} \end{array}\right)\in M_{n\times (N\cdot L)}\left(R\right)\\ & =\left(\begin{array}{ccccccc} X^{1} & | & X^{2} & | & \cdots & | & X^{N} \end{array}\right) \end{array}$$

where $M_{n\times (N\cdot L)}\left(R\right)$ is the vector space of n×(N·L) matrices over R and N∈N is the number of sensors. It is worth noting that each row vector $X\left(i,:\right)\in R^{N\cdot L},\;i=1,\ldots ,n$ of matrix X in Equation (1) represents the measurements from all of the sensors at a given experimental trial. Similarly, each column vector $X\left(:,j\right)\in R^{n},\;j=1,\ldots ,N\cdot L,$ contains measurements from one sensor at one specific time instant in the whole set of experimental trials.

As stated before, one of the goals of PCA is to eliminate the redundancies in the original data. This objective is achieved through a linear transformation orthogonal matrix:

$$\begin{array}{c} P\in M_{\left(N\cdot L)\times (N\cdot L\right)}\left(R\right) \end{array}$$

that is used to transform or project the original data matrix X in Equation (1) according to the matrix product:

$$\begin{array}{c} T=\mathrm{XP}\in M_{n\times (N\cdot L)}\left(R\right) \end{array}$$

where the resulting matrix T has a diagonal covariance matrix.

#### 2.1.2. Normalization: Group Scaling

Since the data in matrix X come from several sensors and could have different magnitudes and PCA is not invariant to scale, a preprocessing stage must be applied to rescale the data. This normalization is based on the mean of all measurements of the sensor at the same time instant and the standard deviation of all measurements of the sensor. In this sense, for k=1,…,N, we define:

(2) $$\begin{array}{cc} \mu_{j}^{k} & =\frac{1}{n}\sum_{i=1}^{n}x_{ij}^{k},\;j=1,\ldots ,L, \end{array}$$

(3) $$\begin{array}{cc} \mu^{k} & =\frac{1}{nL}\sum_{i=1}^{n}\sum_{j=1}^{L}x_{ij}^{k}, \end{array}$$

(4) $$\begin{array}{cc} \sigma^{k} & =\sqrt{\frac{1}{nL}\sum_{i=1}^{n}\sum_{j=1}^{L}{\left(x_{ij}^{k}-\mu^{k}\right)}^{2},} \end{array}$$

where $\mu_{j}^{k}$ is the mean of the measures placed at the same column, that is the mean of the *n* measures of sensor *k* in matrix $X^{k}$ at time instants $\left(j-1\right)\Delta$ seconds; $\mu^{k}$ is the mean of all of the elements in matrix $X^{k}$, that is the mean of all of the measures of sensor *k*; and $\sigma^{k}$ is the standard deviation of all of the measures of sensor *k*. Then, the elements $x_{ij}^{k}$ of matrix X are scaled to define a new matrix $\overset{ˇ}{X}$ as:

(5) $$\begin{array}{cc} {\overset{ˇ}{x}}_{ij}^{k} & :=\frac{x_{ij}^{k}-\mu_{j}^{k}}{\sigma^{k}},\;i=1,\ldots ,n,\;j=1,\ldots ,L,\;k=1,\ldots ,N. \end{array}$$

For the sake of simplicity, the scaled matrix $\overset{ˇ}{X}$ is renamed again as X. One of the properties of the scaled matrix X is that it is mean-centered [29]. Consequently, the covariance matrix of X can be defined and computed as:

(6) $$\begin{array}{c} C_{X}=\frac{1}{n-1}X^{T}X\in M_{\left(N\cdot L)\times (N\cdot L\right)}\left(R\right). \end{array}$$

The subspaces in PCA are defined by the eigenvectors and eigenvalues of the covariance matrix as follows:

(7) $$C_{X}P=P\Lambda$$

where the columns of $P\in M_{\left(N\cdot L)\times (N\cdot L\right)}\left(R\right)$ are the eigenvectors of $C_{X}$ and are defined as the principal components. The diagonal terms of matrix $\Lambda \in M_{\left(N\cdot L)\times (N\cdot L\right)}\left(R\right)$ are the eigenvalues $\lambda_{i},\;i=1,\ldots ,N\cdot L,$ of $C_{X}$, whereas the off-diagonal terms are zero, that is,

(8) $$\begin{array}{cc} \Lambda_{ii} & =\lambda_{i},\;i=1,\ldots ,N\cdot L \end{array}$$

(9) $$\begin{array}{cc} \Lambda_{ij} & =0,\;i,j=1,\ldots ,N\cdot L,\;i\neq j \end{array}$$

The goal of principal component analysis is two-fold; on the one hand, to eliminate the redundancies of the original data. This is achieved by transforming the original data through the projection defined by matrix P in Equation (7). On the other hand, the second goal is to reduce the dimensionality of the dataset X. This second objective is achieved by selecting only a limited number ℓ<N·L of principal components related to the *ℓ* highest eigenvalues. In this manner, given the reduced matrix:

(10) $$\begin{array}{c} \hat{P}=\left(p_{1}|p_{2}\left|\cdots \right|p_{\ell }\right)\in M_{N\cdot L\times \ell }\left(R\right), \end{array}$$

matrix $\hat{T}$ is defined as:

(11) $$\begin{array}{c} \hat{T}=X\hat{P}\in M_{n\times \ell }\left(R\right). \end{array}$$

#### 2.1.3. Projection of New Data onto the PCA Model

The current structure to inspect is excited by the same signal as the one that excited the healthy one in Section 2.1.1. Therefore, when the measures are obtained from N∈N sensors during (L−1)Δ seconds and ν∈N experimental trials, a new data matrix Y is constructed as in Equation (1):

(12) $$\begin{array}{cc} Y & =\left(\begin{array}{ccccccc} \begin{array}{cccc} y_{11}^{1} & y_{12}^{1} & \cdots & y_{1L}^{1}\\ \vdots & \vdots & \ddots & \vdots \\ y_{i1}^{1} & y_{i2}^{1} & \cdots & y_{iL}^{1}\\ \vdots & \vdots & \ddots & \vdots \\ y_{v1}^{1} & y_{v2}^{1} & \cdots & y_{vL}^{1} \end{array} & | & \begin{array}{ccc} y_{11}^{2} & \cdots & y_{1L}^{2}\\ \vdots & \ddots & \vdots \\ y_{i1}^{2} & \cdots & y_{iL}^{2}\\ \vdots & \ddots & \vdots \\ y_{v1}^{2} & \cdots & y_{vL}^{2} \end{array} & | & \begin{array}{c} \cdots \\ \ddots \\ \cdots \\ \ddots \\ \cdots \end{array} & | & \begin{array}{ccc} y_{11}^{N} & \cdots & y_{1L}^{N}\\ \vdots & \ddots & \vdots \\ y_{i1}^{N} & \cdots & y_{iL}^{N}\\ \vdots & \ddots & \vdots \\ y_{v1}^{N} & \cdots & y_{vL}^{N} \end{array} \end{array}\right)\in M_{\nu \times (N\cdot L)}\left(R\right) \end{array}$$

It is worth noting, at this point, that the natural number ν (the number of rows of matrix Y) is not necessarily equal to *n* (the number of rows of X), but the number of columns of Y must agree with that of X; that is, in both cases, the number *N* of sensors and the number of time instants *L* must be equal.

Before the collected data arranged in matrix Y are projected into the new space spanned by the eigenvectors in matrix P in Equation (7), the matrix has to be scaled to define a new matrix $\overset{ˇ}{Y}$ as in Equation (5):

(13) $$\begin{array}{cc} {\overset{ˇ}{y}}_{ij}^{k} & :=\frac{y_{ij}^{k}-\mu_{j}^{k}}{\sigma^{k}},\;i=1,\ldots ,\nu ,\;j=1,\ldots ,L,\;k=1,\ldots ,N, \end{array}$$

where $\mu_{j}^{k}$ and $\sigma^{k}$ are the real numbers defined and computed in Equations (2) and (4), respectively.

The projection of each row vector $r^{i}=\overset{ˇ}{Y}\left(i,:\right)\in R^{N\cdot L},i=1,\ldots ,\nu$ of matrix $\overset{ˇ}{Y}$ into the space spanned by the eigenvectors in $\hat{P}$ is performed through the following vector to matrix multiplication:

(14) $$\begin{array}{c} t^{i}=r^{i}\cdot \hat{P}\in R^{\ell }. \end{array}$$

For each row vector $r^{i},i=1,\ldots ,\nu$, the first component of vector $t^{i}$ is called the first score or Score 1; similarly, the second component of vector $t^{i}$ is called the second score or Score 2, and so on.

### 2.2. Machine Learning

Machine learning has revolutionized the way that complex problems have been tackled with the help of computer programs. In the incessant and relentless pursuit of the best tools for data analysis, machine learning has been highlighted for its capability for providing a quite remarkable set of strategies for pattern recognition. More precisely, when a deterministic mathematical model is difficult to define and data have, at first glance, no correlation, these pattern recognition techniques are generally able to find some kind of relationship. Machine learning strategies and bio-inspired algorithms allow avoiding this difficulty through mechanisms designed to find the answer by themselves. In SHM or related areas, it is possible to find some applications about how machine learning has been used to detect problems such as breaks, corrosion, cracks, impact damage, delamination, disunity and breaking fibers (some pertinent to metals and the others to composite materials) [30]. In addition, machine learning has been also used to provide information about the future behavior of a structure under extreme events such as earthquakes [31].

**Figure 2:**
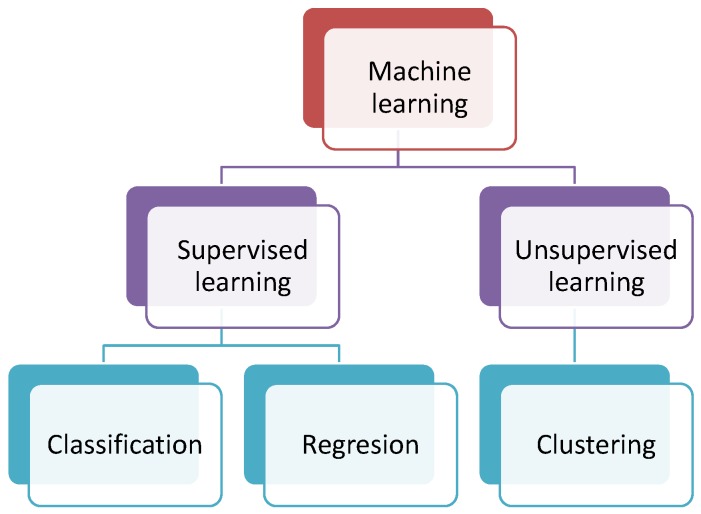
Classification of the machine learning approaches according to the learning.

Depending on how the algorithms are implemented, machine learning can be classified into two main approaches: unsupervised and supervised learning. In the first case, the information is grouped and interpreted using uniquely the input data. However, to perform the learning task in the second case, information about the output data is required. Figure 2 shows this classification and includes information about the kind of tasks that can be performed: clustering, classification and regression.

This paper is focused on the use of supervised learning approaches and, particularly, in the use of nearest neighbor classification, decision trees and support vector machines (SVM). A brief description of the nearest neighbor pattern classification, decision tress and support vector machines is introduced in the following subsections.

#### 2.2.1. Nearest Neighbor Pattern Classification

The nearest neighbor (NN) is a simple nonparametric and highly efficient technique [32] that has been used in several areas such as pattern recognition, ranking models or text categorization and classification for big data [33,34], just to name a few. One of the most used algorithms in machine learning applications is the *k*-NN, also known as *k*-nearest neighbors. *k*-NN stands out due to its simplicity and the excellent results obtained when this technique is applied to diverse problems [35]. This algorithm works by using an input vector with the *k* closest training samples in the feature space. To perform the classification, the algorithm identifies the most common class among the *k* nearest neighbors. The algorithm requires a training to define the neighbors based on the distance from the test sample and a testing step to determine the class to which this test sample belongs [35].

The number of neighbors can be changed to adjust the *k*-NN algorithm. In this sense, for instance, the use of one neighbor is known as fine *k*-NN, and a coarse *k*-NN uses 100 neighbors. Many neighbors can be time consuming to fit. There are six different *k*-NN classifiers available in MATLAB that can be used to classify data [36], and these classifiers are based on different distances. Some of them—fine, medium and coarse *k*-NN algorithms—make use of the Euclidean distance to determine the nearest neighbors. According to MATLAB, each classifier works as follows [35]:

- Fine *k*-NN: a nearest neighbor classifier that makes finely-detailed distinctions between classes with the number of neighbors set to one.
- Medium *k*-NN: a nearest neighbor classifier with fewer distinctions than a fine *k*-NN with the number of neighbors set to 10.
- Coarse *k*-NN: a nearest neighbor between classes, with the number of neighbors set to 100.
- Cosine *k*-NN: a nearest neighbor classifier that uses the cosine distance metric. The cosine distance between two vectors *u* and *v* is defined as:
  $$\begin{array}{c} 1-\frac{u\cdot v}{\left|u|\cdot |v\right|}, \end{array}$$
  that is, one minus the ratio of the inner product of *u* and *v* over the product of the norms of *u* and *v*.
- Cubic *k*-NN: a nearest neighbor classifier that uses the cubic distance metric. The cubic distance between two *n*-dimensional vectors *u* and *v* is defined as:
  $$\begin{array}{c} \sqrt[{3}]{\sum_{i=1}^{n}{\left|u_{i}-v_{i}\right|}^{3}}. \end{array}$$
- Weighted *k*-NN: a nearest neighbor classifier that uses distance weighting. The weighted Euclidean distance between two *n*-dimensional vectors *u* and *v* is defined as:
  $$\begin{array}{c} \sqrt{\sum_{i=1}^{n}w_{i}{\left(x_{i}-y_{i}\right)}^{2}}, \end{array}$$
  where $0<w_{i}<1$ and $\sum_{i=1}^{n}w_{i}=1$.

*k*-NN has been used successfully in fault detection for gas sensor arrays [33], classification for big data [37], fault detection and classification for high voltage DC transmission lines [35] and traffic state prediction [38], among others.

#### 2.2.2. Decision Trees

These machine learning methods are non-parametric computationally-intensive methods [39] that can be applied to regression and classification problems and can work with datasets with a large amount of cases and variables [40]. In general, these methods work by segmenting the predictor space into a number of simple regions.

Some of the advantages and disadvantages of these methods are:

- Compared with other machine learning methods, trees are simple and easy to understand.
- Decision trees use different methods and can be combined to obtain a single prediction.
- The combination of different trees usually produces better results.
- Because of its simplicity, more elaborated methods can produce better results in classification and regression tasks.

Different techniques have been proposed, among them bagging or bootstrap and boosting stand out. In the first, many bootstrap samples are obtained from the data; some prediction method is applied to each bootstrap sample; and then, the results are combined. In the regression case, the combination of the results is performed by averaging, while simple voting is used for classification [39]. Bagging is a committee-based approach that uses a prediction method and the weighted average of the results to obtain an overall prediction.

#### 2.2.3. Support Vector Machines

Support vector machines (SVM) are supervised methods commonly used for regression and classification tasks [41]. In the case of classification, SVM creates a maximum-margin hyperplane that separates all data points from different classes. The support vectors corresponds to the data points that are closest to the separating hyperplane.

## 3. Damage Classification Methodology

In an automated structural health monitoring system, the monitoring system should decide autonomously whether the host structure is damaged or not [7]. With this purpose in mind, this work proposes a damage classification methodology for structures that are subjected to temperature changes. This strategy is described in the following sections.

### Data Acquisition System

**Figure 3:**
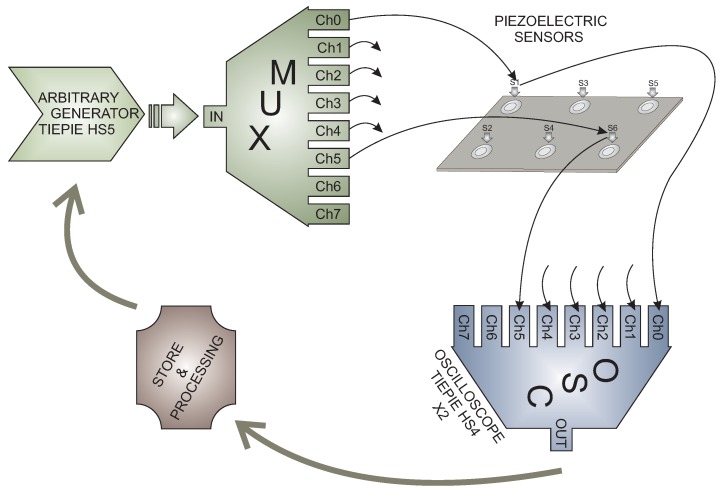
Representation of the structural health monitoring (SHM) system.

The methodology uses data from a structure instrumented with a piezoelectric transducer network. Figure 3 shows the scheme of the data acquisition system where it can be observed that the sensors are attached to the structure. Each piezoelectric transducer (PZT) can operate as an actuator or as a sensor in several actuation phases. Each actuation phase defines a particular piezoelectric as an actuator, and therefore, this PZT excites the structure with a given excitation signal. The rest of the PZT acts as a sensor in such a way that the measured and discretized signals are organized as described in Section 2.1.1, ready to be used in the classification algorithms. The number of actuation phases corresponds to the number of piezoelectric transducers installed in the structure.

**Figure 4:**
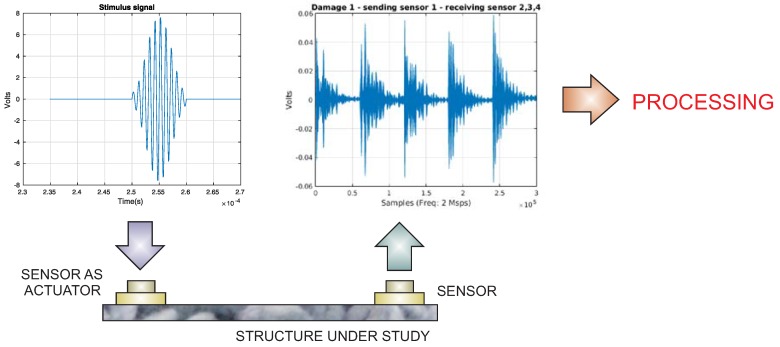
Signal excitation.

The use of piezoelectric transducers is justified by the fact that this kind of sensor is able to produce Lamb waves through the excitation of an actuator with an arbitrary waveform, as is represented in Figure 4. At the same time, the propagated wave, with information about the state of the structure at different locations, is collected by the rest of sensors, since piezoelectric transducers can sense the propagated lamb waves and the information can be captured by a digitizer card. The proposed SHM system is able to work with an arbitrary wave generator, a digitizer card, a personal computer (PC) and a multiplexor card to select the actuator/sensors in each actuation phase.

**Figure 5:**
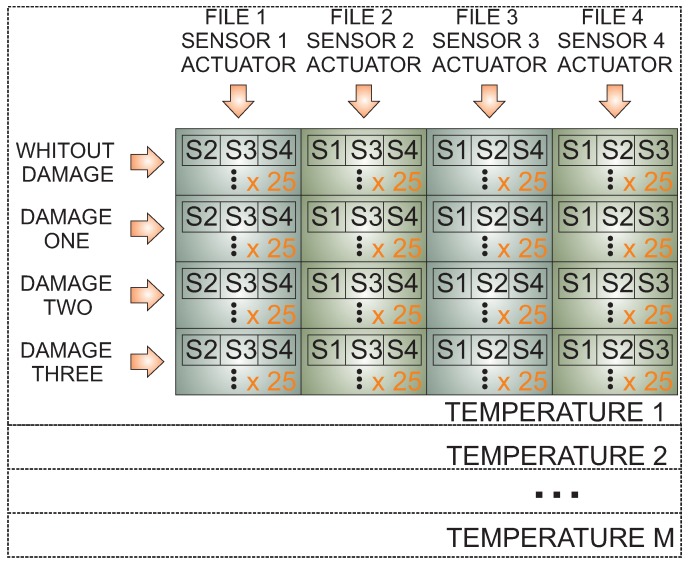
Data organization per each temperature.

Figure 5 can be used as a schematic representation of the way data are collected and organized, also showing the way the data are collected and organized for each actuation phase. That is, in the actuation Phase 1, Sensor 1 is used as an actuator, and the measured data from the sensors 2,3,…,N is captured and organized. In the example represented in Figure 5, four piezoelectric transducers are used. The procedure, however, is identical in the case of a different number of piezoelectric transducers.

To include the effect of the temperature in the proposed methodology, data from each temperature has to be considered. In this specific case, the system requires data from all of the structural states (without damage, Damage 1, Damage 2 and Damage 3, for instance) to consider in the classification under a wide range of temperatures ($T_{1},\ldots ,T_{M}$). Each temperature defines a submatrix where the rows represents the different structural states and columns the different actuation phases. Figure 5 represents an example with four structural states (no damage, Damage 1, Damage 2 and Damage 3), four actuation phases and *M* temperatures.

**Figure 6:**
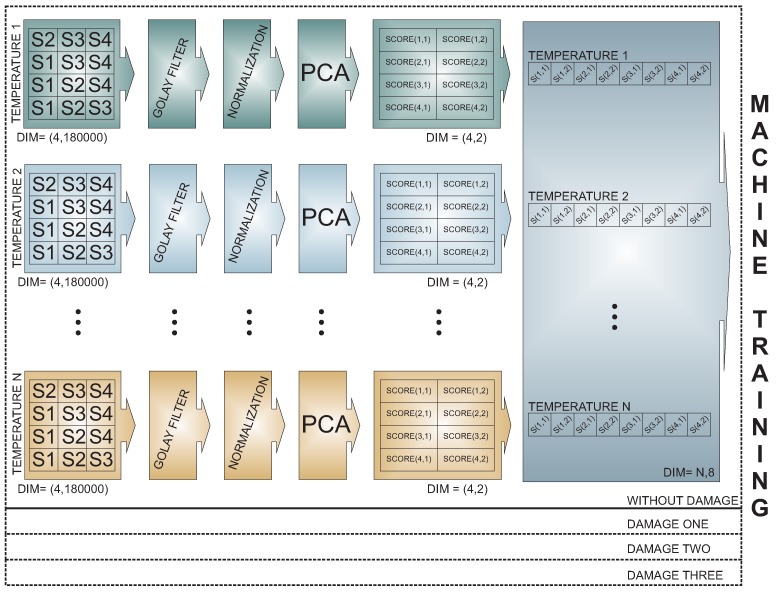
Methodology and training machines.

After the organization of the data for each actuation phase, the methodology considers two general steps or phases: (a) training; and (b) testing. During the training step, data from the healthy or pristine structure subjected to different temperatures are used to train the machines. Figure 6 includes a representation of the steps that are needed between the data acquisition and the machine training. These steps include a data normalization as in Section 2.1.2 [42,43] and principal component analysis (PCA). In this case, we consider the projection onto the first two principal components (scores) as the input to train the machine. The trained machine is then considered as the pattern.

**Figure 7:**
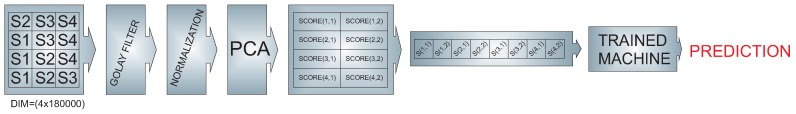
Methodology for prediction.

The testing step considers the use of new data coming from the structure to be diagnosed in an unknown state. These collected data are pre-processed in an identical manner as the data collected from the pristine structure. This means that these data are normalized, and then, the normalized data are projected onto the first two principal component of the PCA model. Finally, the pattern defined by the trained machine will be able to predict the current state of the structure, as depicted in Figure 7.

## 4. Experimental Setup and Results

In this paper, two specimens (structures) are used to explore and demonstrate the feasibility of the structural health monitoring system, for damage identification in structures subjected to temperatures changes, introduced in Section 3. These two specimens are:

(i) an aluminum plate with four piezoelectric transducers; and
(ii) a composite plate of carbon fiber polymer with six piezoelectric transducers.

These two specimens differ in the kind of material, size and number of sensors used. In both cases, the same data acquisition sub-system is used as is represented in Figure 3.

### 4.1. First Specimen: Aluminum Plate

**Figure 8:**
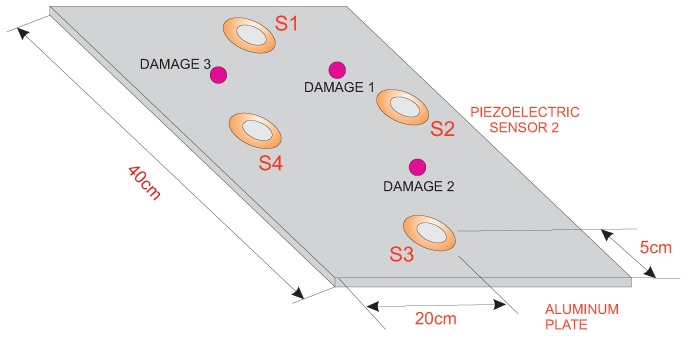
Aluminum plate instrumented with four piezoelectric sensors.

The first specimen that we consider in this paper is an aluminum plate with an area of 40 cm × 40 cm that is instrumented with four piezoelectric sensors. The distribution of the piezoelectric transducers and the size and geometry of the specimen are shown in Figure 8. This figure also indicates the location of the three damages that are presented in the structure.

**Figure 9:**
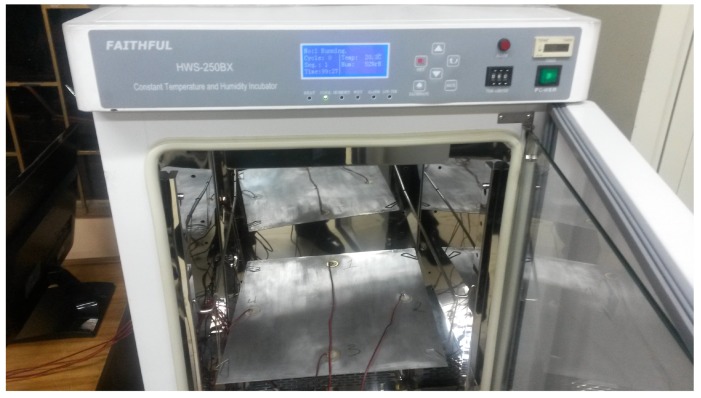
Aluminum plate inside the climate chamber (Faithful HWS-250BX).

To test the structure under different environmental conditions and, more precisely, under different temperatures temperatures, an incubator or climatic chamber (Faithful, Model HWS-250BX) is used to apply these variations. A picture of the aluminum plate inside the chamber can be found in Figure 9.

The experimental setup includes testing with five different temperatures:

- $T_{1}=10^{\circ };$
- $T_{2}=20^{\circ };$
- $T_{3}=30^{\circ };$
- $T_{4}=40^{\circ };$ and
- $T_{5}=45^{\circ }$.

For each one of these five temperatures, data from each structural state are captured. In this case, we have considered four different structural states:

- no damage (healthy or pristine structure);
- Damage 1;
- Damage 2; and
- Damage 3.

**Figure 10:**
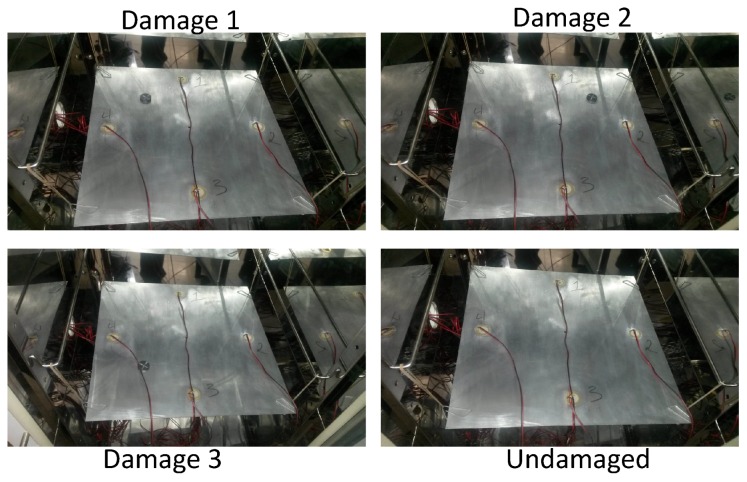
The plate in the climate chamber.

The location of the three damages that are presented in the structure can be found in Figure 8. Figure 10 shows the experimental setup for the four different structural states. As can be observed, the damage is simulated in the structure, in a non-destructive way, as an added mass. The added mass is a magnet, which is attached in both sides of the structure to ensure the position; because aluminum is non-magnetic, the main aspect of this kind of damage is to change the properties of the structure and produce changes in the propagated wave.

It is well known that temperature changes affect the overall behavior of the Lamb waves. More precisely, these changes affect how the Lamb waves propagate, the velocity of the wave over the surface [44] and even the adhesive used to fix the sensors [45]. A very detailed study on the temperature effects in ultrasonic Lamb waves can be found in the work by Lanza di Scalea and Salamone [46]. One of the main conclusions of this work is that the temperature has an imperceptible effect on the wavelength tuning points and a pronounced effect on the response amplitude. In this sense, the goal of the proposed methodology is to include these variations in the structural health monitoring system to avoid false alarms and missing faults in the identification process.

**Figure 11:**
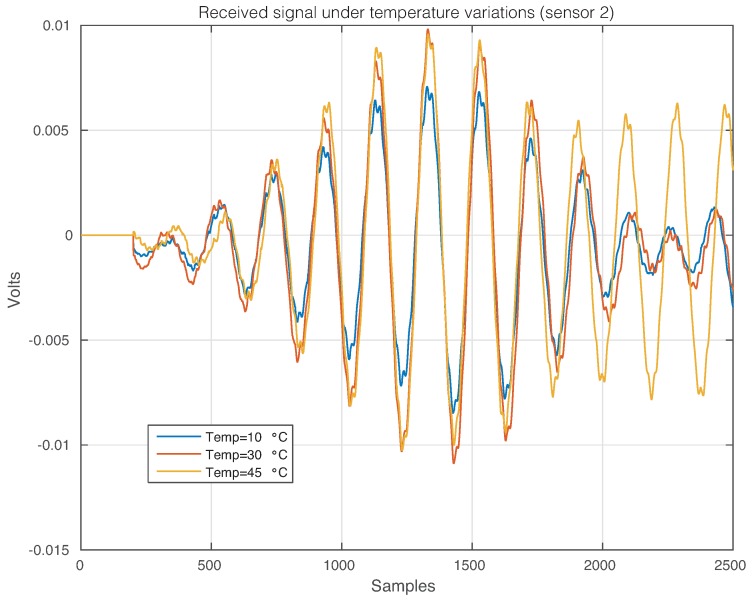
Signal that is received by Sensor 2 when the first sensor is used as an actuator.

**Figure 12:**
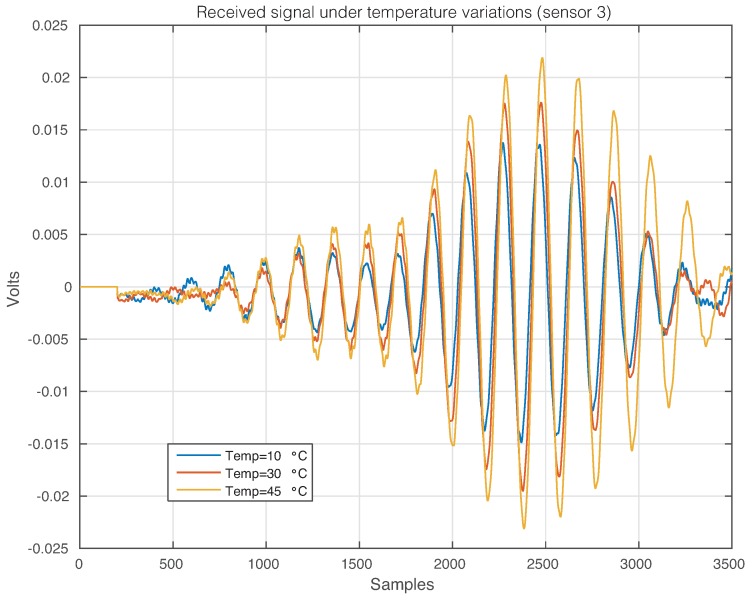
Signal that is received by Sensor 3 when the first sensor is used as an actuator.

**Figure 13:**
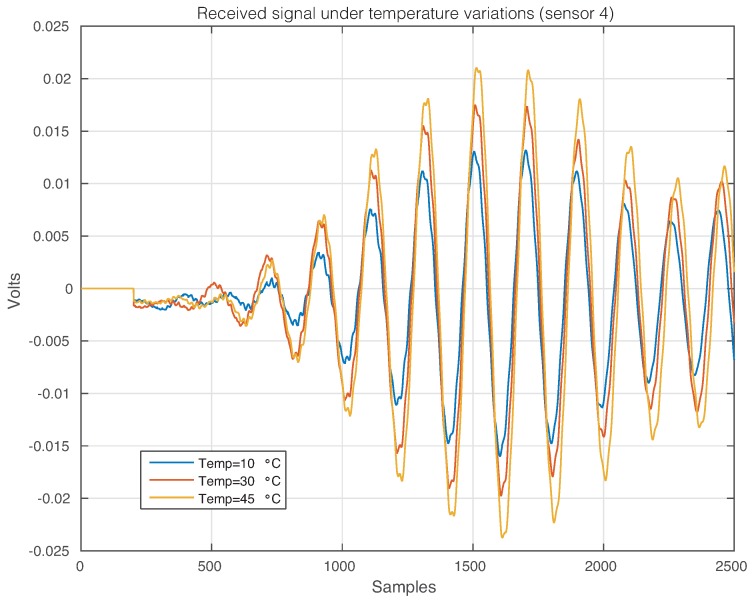
Signal that is received by Sensor 4 when the first sensor is used as an actuator.

The effect of the temperature changes can be perfectly illustrated in Figure 11, where the time-history signal that is received by Sensor 2 when the first sensor is used as an actuator is depicted, for three different temperatures. From this figure, it is possible to observe that changes in the temperature imply changes in the waveforms. More precisely, variations in the phase and amplitude can be easily detected, but some other and more complex changes can also be present [47]. Figure 12 and Figure 13 show the signals received by Sensors 3 and 4, respectively, when the first piezoelectric transducer is used as an actuator. Inspecting both figures, as in Figure 11, there is a clear effect of the temperature with respect to the phase and amplitude of the measured signals. It is worth keeping in mind that the distance between Sensors 1–2 and Sensors 1–4 is equal, while the distance between Sensors 1–3 is relatively larger.

**Figure 14:**
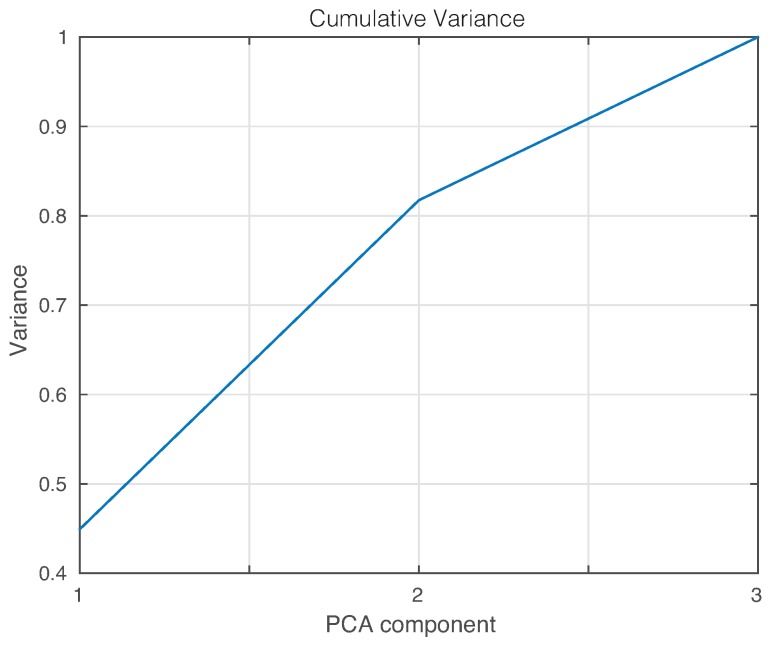
Cumulative contribution rate of variance for the principal components.

The feature vector that is used to train and to test the machines is formed by the projections or scores of the original data into the PCA model created as described in Section 2.1.1. In general, the number of scores that have to be considered depends on the cumulative contribution of variance that it is accounted for. More precisely, the *i*-th score is related to the eigenvector $p_{i}$, defined in Equation (10), and the eigenvalue $\lambda_{i}$, in Equation (8); the cumulative contribution rate of variance accounting for the first σ∈N scores is defined as:

$$\begin{array}{c} \frac{\sum_{i=1}^{\sigma }\lambda_{i}}{\sum_{i=1}^{\ell }\lambda_{i}}, \end{array}$$

where ℓ∈N is the number of principal components. In this sense, the cumulative contribution of the first three scores is depicted in Figure 14. In this experimental setup, we will use the first two principal components that account for more than 80% of the variance. A priori, better results should be obtained if we use as many principal components as possible. However, in some cases, as reported in [48,49], less principal components may lead to more accurate results.

**Figure 15:**
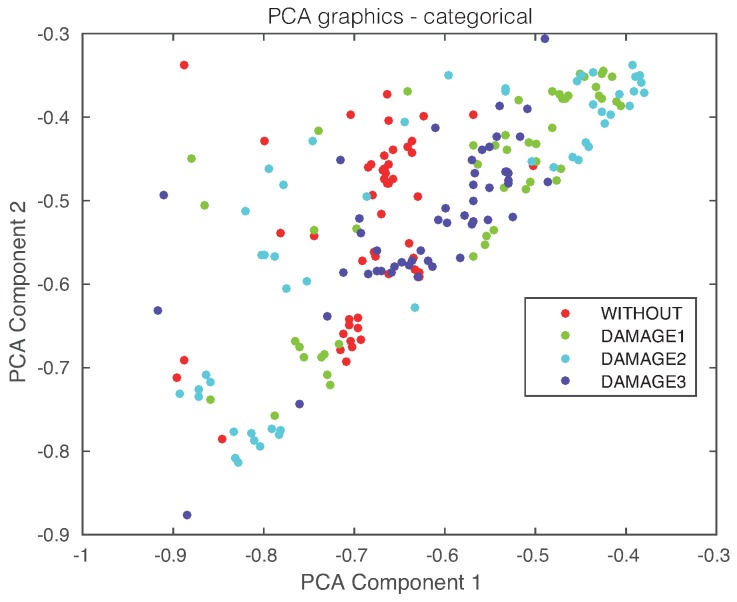
First principal component versus second principal component in the aluminum plate described in Section 4.1.

In a standard application of the principal component analysis strategy in the field of structural health monitoring, the scores allow a visual grouping or separation [50]. In some other cases, as in [51], two classical indices can be used for damage detection, such as the *Q* index (also known as square prediction error (SPE)) and Hotelling’s $T^{2}$ index. In this case, however, it can be noticed in Figure 15, where the projection onto the two first principal components of samples coming from the pristine structure and the structure with damage, subjected to temperatures changes are plotted, that a visual grouping, clustering or separation cannot be performed. To solve this problem, several strategies have been applied in the literature. Some of these procedures are related to univariate or multivariate statistical hypothesis testing [29,48,49]. In this work, an exhaustive number of machine learning approaches is used. This way, some orientations can be presented on the most convenient schemes.

**Figure 16:**
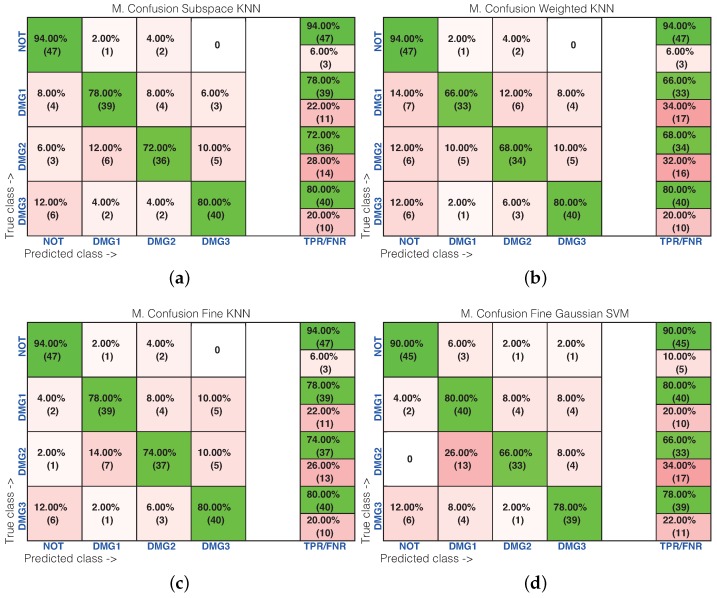
Confusion matrix using: (**a**) subspace *k*-NN; (**b**) weighted *k*-NN; (**c**) fine *k*-NN; and (**d**) fine Gaussian SVM.

**Figure 17:**
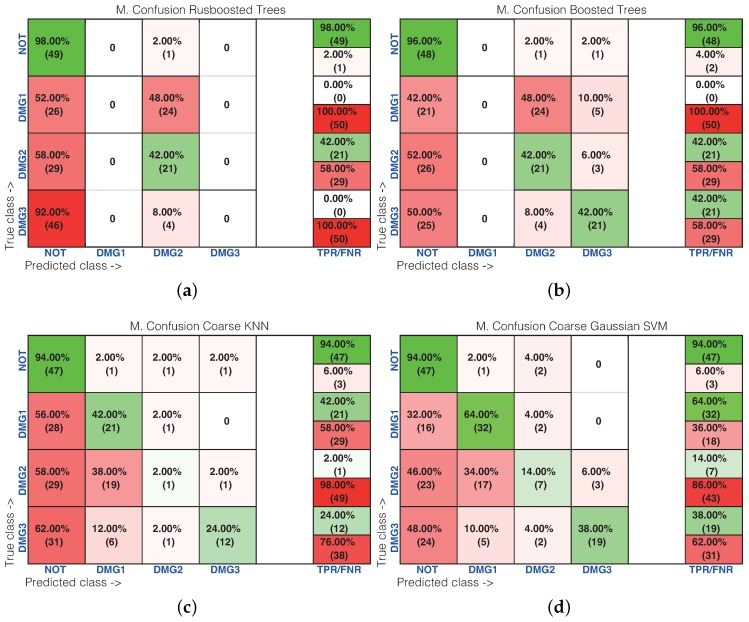
Confusion matrix using: (**a**) rusboosted trees; (**b**) boosted trees; (**c**) coarse *k*-NN; and (**d**) coarse Gaussian SVM.

Table 1 shows the results of the damage identification obtained with the 20 different machine learning strategies. To this goal, the Classification Learner of MATLAB was used. The columns in Table 1 correspond to the percentage of correct decisions for the healthy structure and the structure with Damages 1, 2 and 3. The detailed results can be found in Figure 16 and Figure 17, where the machines with the best and worst performance have been considered, respectively. More precisely, in the subspace *k*-NN classifier, 162 cases have been correctly classified out of 200 cases, while in the fine *k*-NN classifier, this number rises up to 163 cases. Similarly, with respect to the weighted *k*-NN and the fine Gaussian SVM classifiers, 154 and 157 cases have been correctly classified. This represents 77–82% of correct decisions. It is worth noting in these four cases that we have considered that the structure with no damage is correctly classified in more than 90% of cases. Similarly, the structure with damage is confused with the structure with no damage in just a few cases. For instance, in the fine Gaussian SVM classifier, eight cases of the structure with damage are identified as healthy, which represents 5.3% out of 150 cases. As stated before, Figure 17 shows the confusion matrix for the machines with the poorest performance. These are: rusboostedtrees, boosted trees, coarse *k*-NN and coarse Gaussian SVM. For instance, in both rusboosted trees and boosted trees, not one of the samples coming from the structure with Damage 1 is correctly classified. However, in these two cases, 49 and 48 cases of the structure with no damage have been correctly classified, out of 50 cases, which represents 98% and 96%, respectively.

### 4.2. Second Specimen: Carbon Fiber Plate

**Figure 18:**
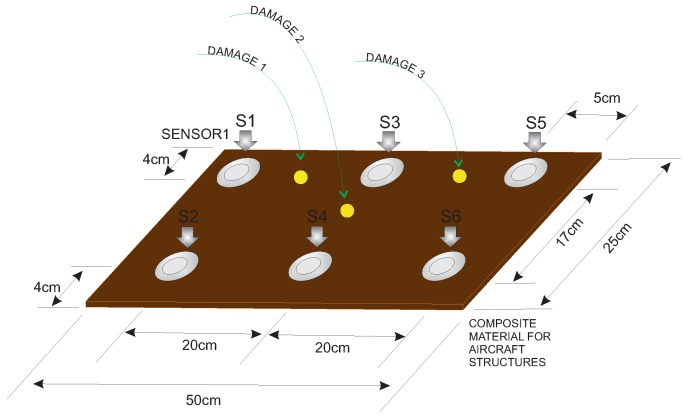
Experimental setup for the composite plate.

The second specimen used for the experimental validation of the approach presented in this paper is a composite plate of carbon fiber polymer with an area of 50 cm × 25 cm and a 2-mm thickness. The plate is instrumented with piezoelectric transducers. Figure 18 shows the dimensions and distribution of the six piezoelectric transducers attached to the structure, as well as the location of the three damages that are presented in the structure.

**Figure 19:**
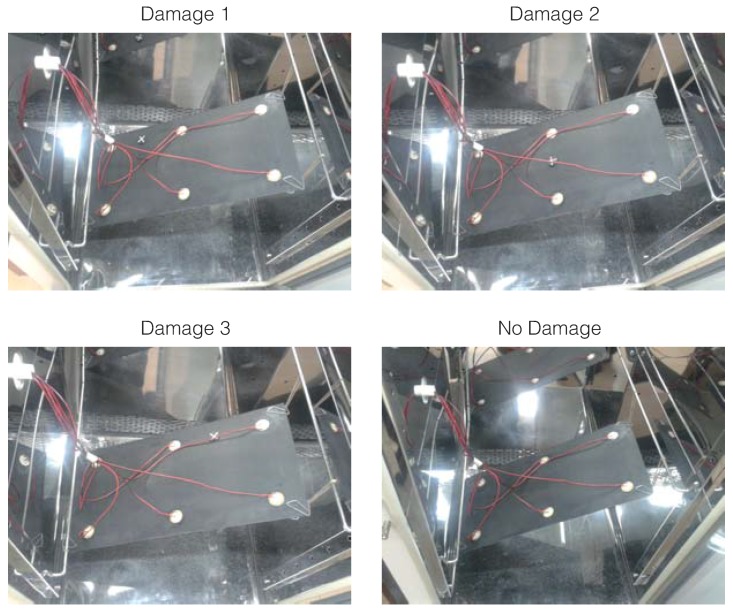
Composite plate in the climatic chamber.

As in the previous experimental setup, to test the structure under different environmental conditions and, more precisely, under different temperatures temperatures, an incubator or climatic chamber (Faithful, Model HWS-250BX) is used to apply these variations. A picture of the composite plate inside the chamber can be found in Figure 19.

The experimental setup includes testing with six different temperatures:

- $T_{1}=0^{\circ };$
- $T_{2}=10^{\circ };$
- $T_{3}=20^{\circ };$
- $T_{4}=30^{\circ };$
- $T_{5}=40^{\circ };$ and
- $T_{6}=45^{\circ }$.

For each one of these six temperatures, data from each structural state are captured. In this case, we have considered four different structural states:

- no damage (healthy or pristine structure);
- Damage 1;
- Damage 2; and
- Damage 3.

**Figure 20:**
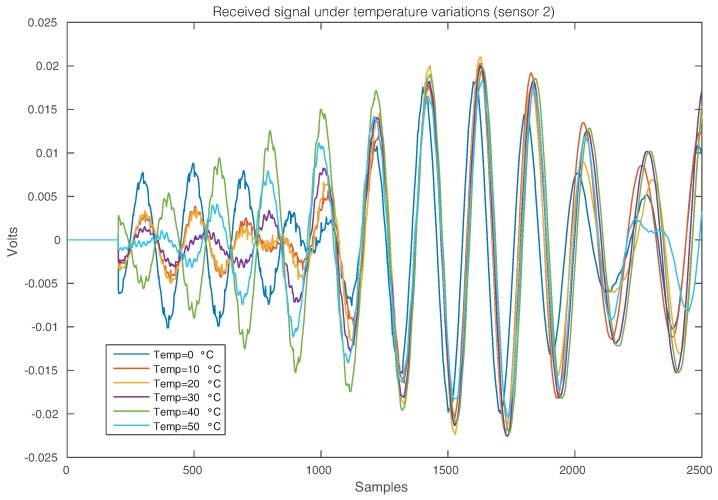
Signal that is received by Sensor 2 when the first sensor is used as an actuator.

The effect of the temperature changes in the composite plate can be perfectly illustrated in Figure 20, where the time-history signal that is received by Sensor 2 when the first sensor is used as an actuator is depicted, for the six different temperatures. As in the previous experimental setup, from this figure, it is possible to observe that changes in the temperature imply changes in the waveforms. More precisely, variations in the phase and amplitude can be easily detected.

**Figure 21:**
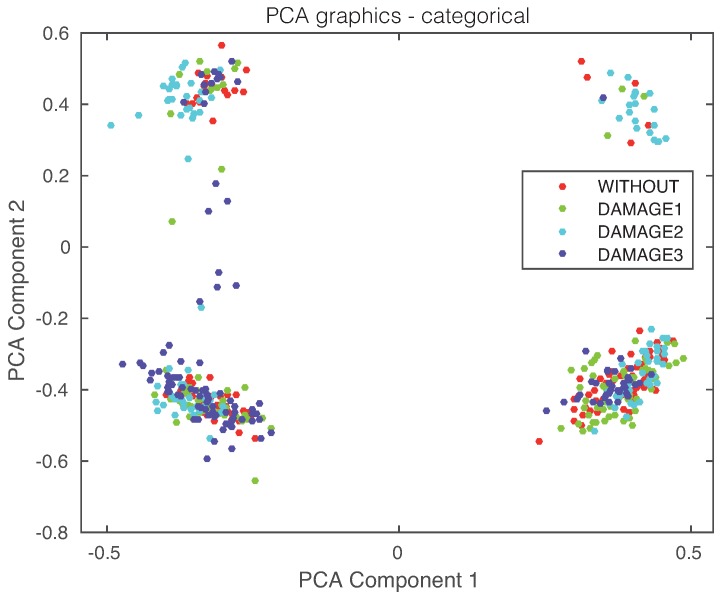
First principal component versus second principal component in the carbon fiber plate described in Section 4.2.

**Figure 22:**
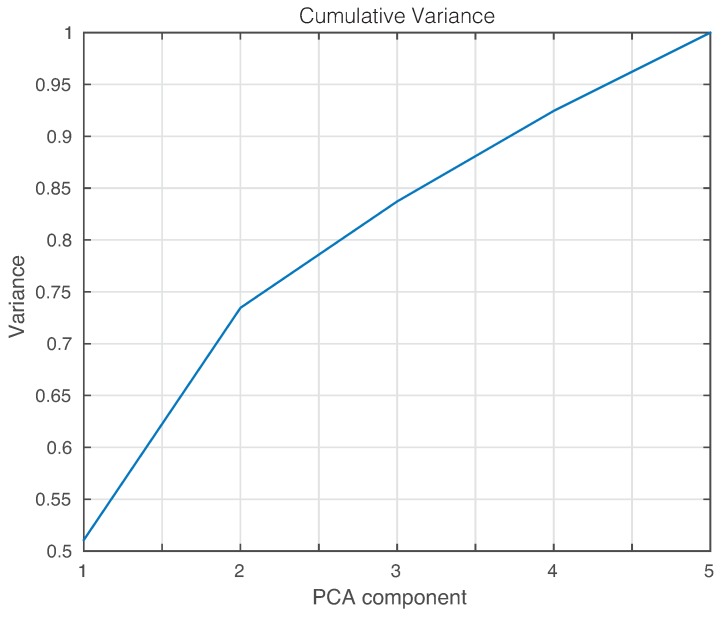
Cumulative variance for the scores of PCA.

Finally, the first principal component versus the second principal component is plotted in Figure 21. It can be observed, again, that a visual grouping, clustering or separation cannot be performed. In this experimental setup, we will use the first three principal components that account for more than 80% of the variance, as can be seen in Figure 22.

**Figure 23:**
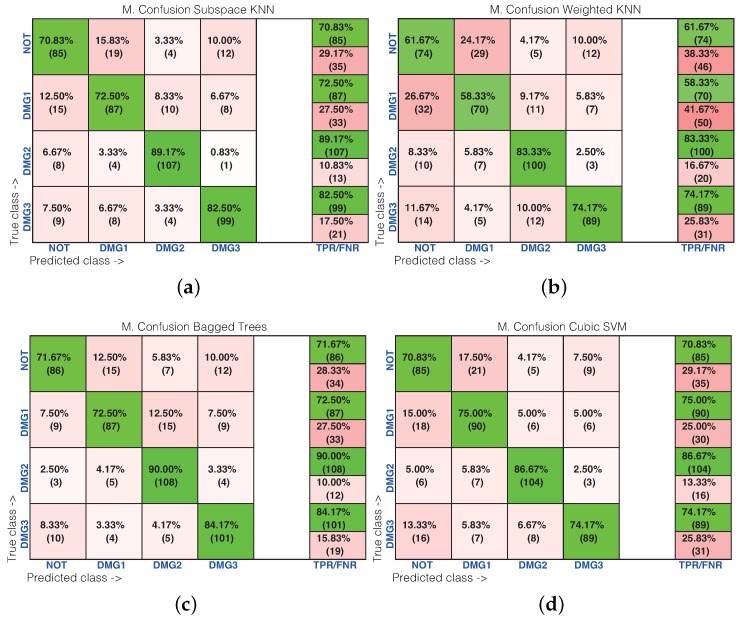
Confusion matrix machines with good behavior. (**a**) Subspace *k*-NN; (**b**) weighted *k*-NN; (**c**) bagged Trees; (**d**) cubic SVM.

**Figure 24:**
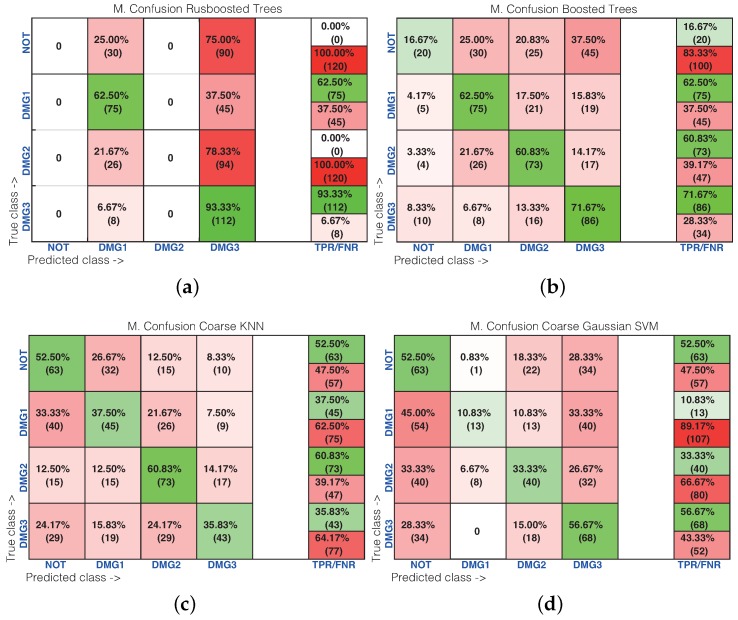
Confusion matrix machines with bad behavior. (**a**) Rusboosted trees; (**b**) boosted trees; (**c**) coarse *k*-NN; (**d**) coarse Gaussian SVM.

Table 2 shows the results of the damage identification in the composite plate obtained with the 20 different machine learning strategies. The columns in Table 2 correspond to the percentage of correct decisions for the healthy structure and the structure with Damages 1, 2 and 3. The detailed results can be found in Figure 23 and Figure 24, where the machines with the best and worst performance have been considered, respectively. More precisely, in the subspace *k*-NN classifier, 378 cases have been correctly classified out of 480 cases, while in the bagged trees classifier, this number rises up to 382 cases. Similarly, with respect to the weighted *k*-NN and the cubic SVM classifiers, 336 and 368 cases have been correctly classified. This represents 70–80% of correct decisions. It is worth noting that in these four cases that we have considered, the structure where Damage 2 is present is correctly classified in more than 83% of cases. Similarly, the structure with damage is confused with the structure with no damage in just a few cases. For instance, in the bagged trees classifier, 22 cases of the structure with damage are identified as healthy, which represents 6.1% out of 360 cases. As stated before, Figure 17 shows the confusion matrix for the machines with the poorest performance. These are: rusboosted trees, boosted trees, coarse *k*-NN and coarse Gaussian SVM. For instance, in rusboosted trees, not one of the samples coming from the healthy structure is correctly classified. However, in this case, 75 cases of the structure with Damage 2 have been correctly classified, out of 120 cases, which represents 75%.

## 5. Concluding Remarks

In this contribution, a structural health monitoring methodology has been developed for damage detection and classification of structures that are subjected to changes in the environmental conditions. The experimental results that have been presented in this work demonstrate that changes in the temperature affect basic damage detection strategies based on principal component analysis; this is because pattern recognition approaches in SHM applications use data from a structure under a established conditions to define a pattern and small changes in the data from the structure as obtained by the variation of temperature produce differences with the pattern and false positive damage detection procedures even it is a healthy structure. In this sense, to overcome the distortion caused by these changing environmental conditions, a more complex SHM strategy has been presented, based on: (i) ultrasonic signals through a piezoelectric sensor network; (ii) principal component analysis; and (iii) pattern recognition based on machine learning approaches, which considers data from different structural states under different temperatures.

According to the experimental results on both an aluminum plate and a composite plate of carbon fiber polymer, subspace *k*-NN and weighted *k*-NN have presented the most accurate results. Besides, for the aluminum plate, fine *k*-NN and fine Gaussian *k*-NN classifiers showed a very good behavior. For the composite plate, bagged trees and cubic SVM were also quite accurate.

Among the classifiers, the ones with the poorest accuracy were rusboosted trees, boosted trees, coarse *k*-NN and coarse Gaussian SVM. The advantages of the developed methodology include: (i) a data-driven analysis that allows the knowledge of the current state of the structure directly from the collected data and without the use of a complex mathematical model; (ii) the reduction of false positives, since data from different temperatures are considered during the training and sensor data fusion to provide a single a more reliable result. One of the disadvantages of the methodology is the big quantity of data required to cover all of the structural states with respect to all of the temperatures. Besides, a new damage can be detected as such, but it cannot be properly classified since there is no information about this particular damage within the pattern.

Since the methodology allows detecting and classifying a damage with data collected from the structure, damage localization can be explored by understanding that a huge quantity of data of damage in different positions of the structure can be used not only for classification, but also for localization if the position of the damage is defined from the beginning in the training process. A variation of this methodology is being explored in other work where machine learning approaches are used for regression.

## Figures and Tables

**Table 1:** Percentage of correct decisions for the healthy structure and the structure with Damage 1, 2 and 3, for the twenty different machine learning strategies (aluminum plate).

Machine Name	Healthy	Damage 1	Damage 2	Damage 3
Medium Tree	66%	76%	70%	56%
Simple Tree	64%	60%	30%	58%
Complex Tree	72%	76%	58%	56%
Linear SMV	70%	60%	26%	60%
Quadratic SVM	78%	70%	56%	70%
Cubic SVM	86%	68%	66%	72%
Fine Gaussian SVM	90%	80%	66%	78%
Medium Gaussian SVM	76%	80%	56%	74%
Coarse Gaussian SVM	94%	64%	14%	38%
Fine *k*-NN	94%	78%	74%	80%
Medium *k*-NN	80%	62%	64%	74%
Coarse *k*-NN	94%	42%	2%	24%
Cosine *k*-NN	84%	58%	78%	72%
Cubic *k*-NN	80%	64%	62%	76%
Weighted *k*-NN	94%	66%	68%	80%
Boosted Trees	96%	0%	42%	42%
Bagged Trees	84%	70%	66%	78%
Subspace Discriminant	56%	44%	32%	46%
Subspace *k*-NN	94%	78%	72%	80%
Rusboosted Trees	98%	0%	42%	0%

**Table 2:** Percentage of correct decisions for the healthy structure and the structure with Damages 1, 2 and 3, for the twenty different machine learning strategies (composite plate).

Machine Name	Healthy	Damage 1	Damage 2	Damage 3
Medium Tree	55.00%	63.33%	60.83%	52.50%
Simple Tree	40.00%	60.00%	63.33%	42.50%
Complex Tree	57.50%	64.17%	75.83%	65.83%
Linear SVM	41.67%	59.17%	45.00%	47.50%
Quadratic SVM	65.83%	73.33%	85.00%	75.50%
Cubic SVM	70.83%	75.00%	86.67%	74.17%
Fine Gaussian SVM	59.17%	64.17%	83.33%	78.33%
Medium Gaussian SVM	55.83%	60.00%	82.50%	63.33%
Coarse Gaussian SVM	52.50%	10.83%	33.33%	56.67%
Fine *k*-NN	63.33%	61.67%	80.00%	70.00%
Medium *k*-NN	65.00%	46.67%	75.00%	63.33%
Coarse *k*-NN	52.50%	37.50%	60.83%	35.83%
Cosine *k*-NN	65.00%	43.33%	79.17%	60.83%
Cubic *k*-NN	59.17%	47.50%	72.50%	60.00%
Weighted *k*-NN	61.67%	58.33%	83.33%	74.17%
Boosted Trees	16.67%	62.50%	60.83%	71.67%
Bagged Trees	71.67%	72.50%	90.00%	84.17%
Subspace Discriminant	33.33%	45.83%	45.00%	55.83%
Subspace *k*-NN	70.83%	72.50%	89.17%	82.50%
Rusboosted Trees	0.00%	62.50%	0.00%	93.33%
